# Editorial: Comparative cranial morpho-physiology applied to domestic and wild animals

**DOI:** 10.3389/fvets.2024.1420168

**Published:** 2024-06-24

**Authors:** Henrique Inhauser Riceti Magalhães, Lucas de Assis Ribeiro, Gerhard Steenkamp, Frank J. M. Verstraete

**Affiliations:** ^1^School of Veterinary Medicine, Cruzeiro do Sul University, São Paulo, Brazil; ^2^School of Veterinary Medicine, Federal University of Uberlândia, Uberlândia, Brazil; ^3^Department of Companion Animal Clinical Studies, Faculty of Veterinary Science, University of Pretoria, Pretoria, South Africa; ^4^Department of Surgical and Radiological Sciences, School of Veterinary Medicine, University of California, Davis, Davis, CA, United States

**Keywords:** anatomy, dentistry, imaging, mandible, morphology, skull, symphysis

With the advancement of specialties within veterinary medicine, dentistry is gaining increasing prominence. The oral cavity, associated tissues, and teeth are essential structures for the health of domestic and wild animals (1–3). The integrity of the mouth is fundamental for maintaining good nutritional status and quality of life (2, 4). In this context, oral care is as an excellent prophylactic and therapeutic strategy, however, it requires a deep anatomical and physiological knowledge (2, 5–8). It is also necessary to consider the specificities of a wide range of species, anticipating them as potential patients (3). Species-specific studies are of great value as they provide a basis for greater clinical-surgical safety by not extrapolating morphological data obtained from different species (9, 10). Furthermore, it is also possible to provide a more detailed and applicable analysis of information obtained from a group of animals or a specific species of interest.

The main goal of this Research Topic was to reinforce the importance of cranial anatomy and physiology as a basis for comparative studies and the improvement of therapeutic approaches in Veterinary Dentistry and Oromaxillofacial Surgery of domestic and wild animals. We present seven peer reviewed articles: one with local anesthetic application, one with surgical application, three with clinical applications, and two with morphophysiological application. Three articles were about dogs, one about cats, one about horses, and two about wild animals (alpaca and dromedary camels). All articles used some form of imaging examination as the main or complementary methodology to conclude their objectives.

The study by Littles et al. used cone-beam computed tomography to describe the topography of the infraorbital canal in relation to the roots of the maxillary fourth premolar tooth in dogs with different types of cranial conformation. One hundred and twenty animals seen for reasons unrelated to the study were studied: 40 mesocephalic dogs, 40 brachycephalic dogs, and 40 dolichocephalic dogs. Variations in the position of the infraorbital canal were observed among different skull types, suggesting surgical implications involving the maxillary fourth premolar tooth. Specifically, the authors recommend that extra precautions be taken in dolichocephalic and brachycephalic dogs due to the increased risk of iatrogenic trauma to the infraorbital canal and orbit during surgical manipulations on the tooth in question. The results demonstrate the importance of specific anatomical knowledge of each patient to avoid surgical complications during dental procedures in dogs.

The article by Proost et al. investigated the morphology of alpaca teeth (*Vicugna pacos*) using nine teeth (maxillary and mandibular molar teeth) extracted from seven specimens that died due to non-dental reasons. The teeth were evaluated using histology and high-resolution micro-computed tomography scanning (μ-CT scanning). The results revealed histological characteristics typical of other species, such as dentin, cementum, and pulp, as well as a rare type of dentin called vasodentin. A high prevalence of resorption lesions, apparently induced by the proximity of the roots of neighboring teeth, was also observed. Further studies are needed to better understand the consequences of this dental morphology and secondary infections in alpacas. This study, however, already provides insights for a better understanding of the etiopathogenesis of some dental diseases in this species.

The third study, conducted by Haseler et al., presents a series of retrospective cases regarding the effectiveness of performing the surgical technique of marsupialization in 12 odontogenic cysts in six boxer dogs. Marsupialization is a minimally invasive method that decompresses the odontogenic cyst and promotes remodeling of alveolar bone and shrinkage of the cyst. High-resolution computed tomography was used as an imaging tool before and during the follow-up of all cases. The results demonstrated a significant reduction in cyst volume after the procedure, which would reduce the risk in subsequent extirpation treatments.

The study by Sterkenburgh et al. explores orthodontic morphometry and dynamic movements of the mandible and temporomandibular joint in horses to better understand the physiology of the incisive occlusal surface. Dental reports from 609 horses over an 18-month period were analyzed. Biomechanical calculations and simulations performed by the authors confirmed that the occlusal surface of the incisor teeth is a plane. Clinical investigations were corroborated by theoretical analysis and mechanical simulations, strengthening the baseline knowledge of the physiological maintenance of the occlusal plane of incisor teeth in horses. The study provides insights into the maintenance of occlusal surfaces during chewing and emphasizes the importance of morphological analysis in early identification and intervention for dental abnormalities in horses.

The first article by Minei et al.(a) describes the physiological variation of clinical mobility of the mandibular symphysis in dogs, seeking associations with breed, body weight, age, sex, and cranial conformation. Radiography was also used in this study. Five hundred and sixty-seven dogs of 95 different breeds were analyzed over a period of 81 months. Most of the animals studied showed zero mobility of the mandibular symphysis, and in cases examined more than once over time, the results did not change. Additionally, the mobility of this joint decreased with increasing age and body weight. Brachycephalic dogs demonstrated a tendency to have a radiographically more divergent mandibular symphysis and greater ventrodorsal joint mobility. The study provides important data on the morphological variation of the mandibular symphysis in dogs, useful for the prevention and treatment planning of mandibular diseases in these animals.

Al Mohamad et al. conducted a topographic and morphometric analysis of the mandibular foramen in dromedary camels (*Camelus dromedarius*) to establish a clinical approach to the region based on a detailed description of anatomical landmarks. Additionally, the course of the inferior alveolar nerve was described. Eight osteometric measurements were used for the mandibular foramen, four heads were dissected to visualize the course of the inferior alveolar nerve within the mandibular canal, and four heads were radiographed as tests for the extraoral approach of local anesthetic block for the inferior alveolar nerve. Despite the need for additional clinical validation of the technique, the article contributes to a better understanding of the specific and applied anatomy of dromedary camels, which is extremely valuable in seeking greater safety in performing veterinary anesthetic and surgical techniques.

Finally, the second article by Minei et al.(b) reports on the normal variation of clinical mobility of the mandibular symphysis in cats, seeking associations with weight, age, sex, breed, and skull morphology. Radiographic aspects were also evaluated. Two hundred and sixteen cats of 15 different breeds were studied for lateromedial and dorsoventral mobility of the joint. Most of the animals studied showed zero dorsoventral mobility and some degree of lateromedial mobility of the mandibular symphysis. An increase in ventrodorsal mobility was associated with increasing age and the presence of resorptive lesions and a decrease in lateromedial mobility with brachycephalic conformation. The article presents clinically relevant findings for the evaluation and treatment of dental and symphyseal disorders in cats.

In summary, we hope that the articles compiled in this Research Topic help to highlight the need for detailed knowledge of cranial anatomy and physiology in veterinary dental practice and may encourage future basic and applied studies in various animal species. The wide variety of approaches, ranging from morphological studies to surgical techniques, reflects the complexity, diversity, and individuality of dental demands in domestic and wild animals. Additionally, the use of different imaging techniques demonstrates the crucial role of technology in complementing classical knowledge and promoting animal health. We believe that these articles provide valuable insights for clinical practice and improve therapeutic approaches in veterinary dentistry and oromaxillofacial surgery.

## Author contributions

HM: Writing – original draft, Validation, Data curation, Conceptualization. LR: Writing – review & editing, Validation, Supervision, Data curation, Conceptualization. GS: Writing – review & editing, Validation, Supervision, Data curation, Conceptualization. FV: Writing – review & editing, Validation, Supervision, Data curation, Conceptualization.
